# The “Under, Over” Technique for Repair of a Peripheral Bucket-Handle Meniscus Tear With Circumferential Compression Stitches

**DOI:** 10.1016/j.eats.2023.03.002

**Published:** 2023-06-19

**Authors:** Sophie E. Lipson, Allyn Morris, Ferdinand J. Chan

**Affiliations:** a Tufts University School of Medicine, Boston, Massachusetts, U.S.A.; b Department of Orthopaedic Surgery, Montefiore Medical Center, Bronx, New York, U.S.A.

## Abstract

The benefits of preserving the meniscus are well-established. Several arthroscopic meniscal repair techniques have been described, such as the inside-out, outside-in, and all-inside. All-inside self-retrieving suture devices can be used to repair vertical, horizontal, and radial tears. However, this technique becomes difficult with large tears, as the jaw of the device cannot reach the peripheral edge of the meniscal tear. We present an all-inside technique using circumferential compression stitches to address large peripheral meniscus tears.

The meniscus functions to increase surface area for force transmission and shock absorption, as well as to provide stability for the knee joint in cases of cruciate ligament deficiency.^1^^,^^2^ Without treatment, meniscal tears can lead to degenerative joint changes.^1^^,^^3^^,^^4^ Meniscal repair has become increasingly common to prevent the early development of osteoarthritis that can occur after subtotal and partial meniscectomies.^4^ Broadly, 3 types of arthroscopic techniques for meniscus repair have been well described: the inside-out, the outside-in, and the all-inside. Although the inside-out remains the gold standard technique for meniscus repair, the all-inside technique is increasingly used for vertical, horizontal, and radial meniscal tears with the advances in suture implant technologies.^2^ However, the traditional all-inside anchor-based technique for lateral meniscus posterior horn tears can place the popliteal neurovascular bundle at risk of injury.^5^^,^^6^ Magnetic resonance imaging studies show that the popliteal artery lies lateral to the midline in approximately 94% of cases.^7^^,^^8^ In addition, traditional all-inside repair of the lateral meniscus places the popliteus tendon at risk of being captured by the anchor, which may lead to tissue irritation or injury.^9^^,^^10^

Self-retrieving suture devices are available to perform the all-inside suture-based technique and reduce risk of injury to the posterior structures. In particular, self-retrieving devices are especially useful for circumferential stitches, which provide anatomic reduction and compression of tears with a high load to failure.^11^, ^12^, ^13^ In order for self-retrieving devices to successfully reduce vertical tears, the jaw of the device must be able to fit between the femoral and tibial condyles as well as reach the peripheral side of the tear. If the meniscal tear is too large, the jaw of the device may have difficulty reaching the peripheral meniscal tissue. We present an all-inside technique to address large these peripheral meniscus tears.

## Surgical Technique (With Video Illustration)

### Patient Setup

After the induction of general anesthesia, the patient is positioned in the supine position. A tourniquet is placed on the operative extremity and the thigh is positioned in a leg holder to allow 90° of knee flexion. Draping is performed in the standard sterile fashion.

### Arthroscopic Access

A standard anterolateral arthroscopic portal is first created, and the anteromedial portal is created under direct arthroscopic visualization. An arthroscopic probe is used to fully evaluate the extent and nature of the meniscus tear. The tear edges are debrided with a combination of a motorized shaver and a round ball rasp. Meniscal trephination is performed with an 18-gauge spinal needle.

### Meniscal Repair

**Fig 1:**
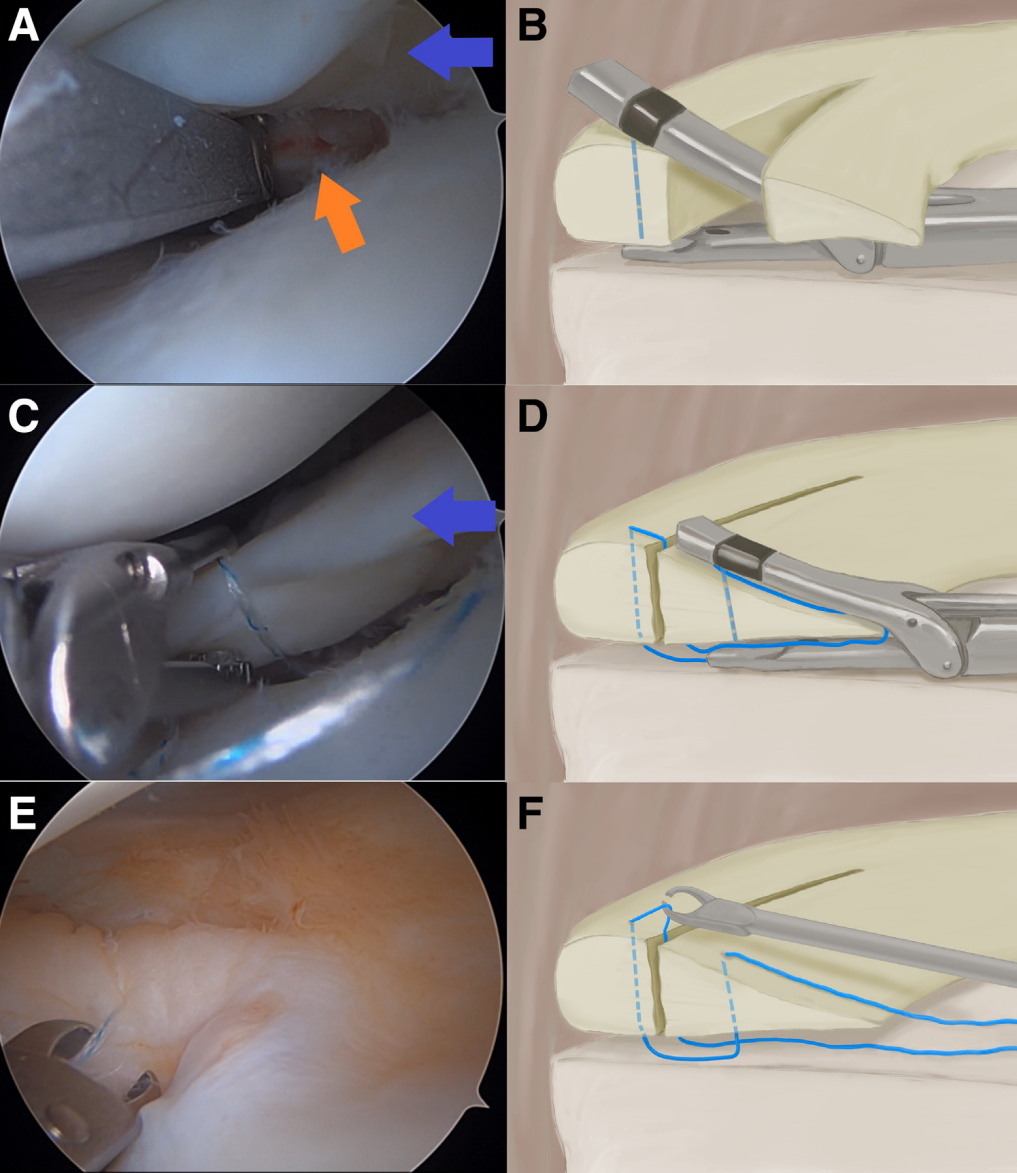
Repair of a right knee bucket-handle lateral meniscus tear. The working portal is initially in the standard anterolateral portal for repair of the posterior horn and transitioned to the standard anteromedial portal for repair of the body and area anterior to the popliteus hiatus. The all-inside self-retrieving suture-based device (NOVOSTITCH PRO device; Smith & Nephew, Andover, MA) is inserted through the working portal and advanced under the central component of the meniscus (blue arrow) until the device is at the site of the tear. The upper jaw is extended through the tear above the femoral surface of the peripheral aspect of the meniscus tear (orange arrow). The first limb of suture is passed through the peripheral side of the tear from the lower to upper jaw of the device, which retains the suture (A, B). The lower jaw of the device is retracted to move the device centrally while the upper jaw retains the first limb of suture. The second limb of suture is then passed through the central side of the tear (blue arrow) to complete the suture construct (C, D). The lower jaw is retracted and the device is removed. The peripheral limb of suture is retrieved using an arthroscopic crab claw superior to both meniscal fragments (E, F).

The repair is performed using the self-retrieving all-inside NOVOSTITCH PRO device (Smith & Nephew, Andover, MA) (Video 1). The device is preloaded with a 2-0 high-strength, nonabsorbable suture before entering the joint through the working portal. The device is advanced in a low-profile configuration with the lower jaw retracted to minimize the risk of traumatic insertion. To maintain the low-profile configuration, the front trigger is squeezed while the device is advanced under the central component of the meniscus tear. Once at the site of the tear, the front trigger is released to lift the upper jaw of the device between the peripheral and central components of the tear. This will position the upper jaw above the femoral surface of the meniscus. The lower jaw is extended below the tibial surface of the meniscus by toggling the lever on the side of the device. The suture is passed through the peripheral side of the tear from the lower jaw to the upper jaw by squeezing the trigger (Fig 1 A and B). The lower jaw can be retracted at this time to allow better maneuverability. The device is moved centrally while the upper jaw retains the first limb of suture. The second limb of suture is then passed through the central side of the tear to complete the suture construct (Fig 1C and D). Care is taken not to pierce the suture limb that is now below the meniscus. The device is removed from the joint. An arthroscopic crab claw is used to retrieve the peripheral suture limb superior to the meniscus (Fig 1 E and F). An arthroscopic knot is tied and cut using a suture cutter. The device can be reloaded with new suture cartridges to place additional circumferential sutures as needed.

**Fig 2:**
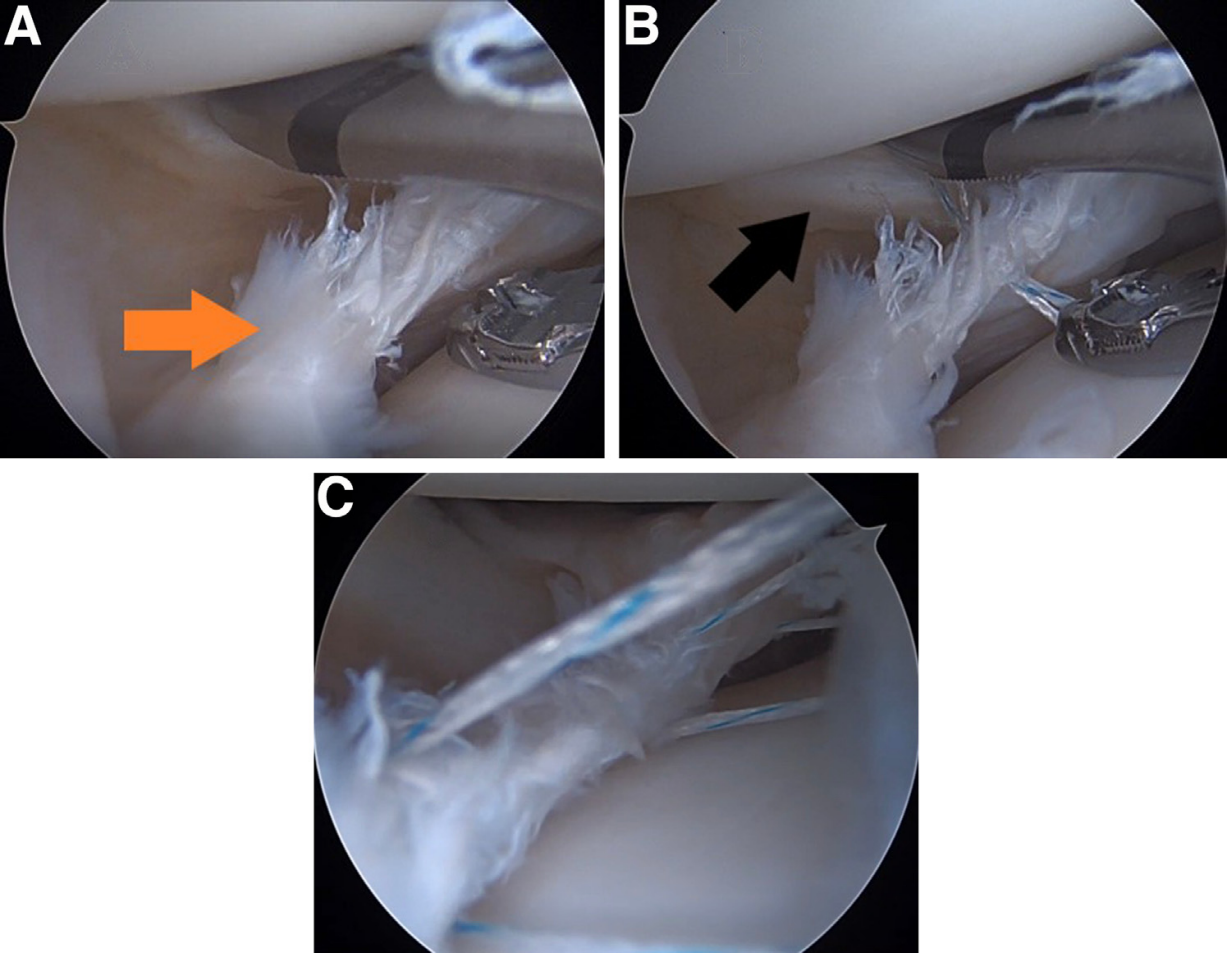
Repair of a right knee bucket-handle lateral meniscus tear. The arthroscope is in the anterolateral portal and NOVOSTITCH PRO device (Smith & Nephew, Andover, MA) is advanced from the anteromedial portal. Minimal meniscal tissue (orange arrow) remains on the peripheral side of the tear anterior to the popliteus tendon (A). Suture is passed and avoids injury to the popliteus tendon (black arrow) (B). Additional sutures are passed to ensure adequate repair (C).

Direct visualization of the peripheral tear can be helpful, especially with small residual meniscal tissue (Fig 2 A and B). This process is repeated until sufficient sutures are in place for adequate meniscus reduction (Fig 2C). It is advantageous to create an accessory portal to house the sutures and tie after passage of all sutures. Premature tying of the sutures can limit access to the peripheral meniscal tissue. After meniscus repair, a 2.0 Kirschner wire is used to make 3 to 6 holes on the lateral femoral notch anterior to the femoral anterior cruciate ligament footprint for biological augmentation.

The patient is braced and weight-bearing is performed as tolerated from postoperative day 0. From postoperative weeks 0-2, range of motion is limited to 0 to 70°. Range of motion is increased to 90° until week 6. From week 6 to 4 months, range of motion is increased to 130°, after which full range of motion is permitted. Hyperflexion and deep squatting are avoided for 4 months.

## Discussion

Bucket-handle meniscus tears should be repaired, when possible, in order to prevent loss of meniscus volume and early development of osteoarthritis. Several studies have reported favorable functional outcomes and patient-reported outcomes after repair of a bucket-handle tear.^14^, ^15^, ^16^ Muench et al.^14^ found that 83.3% of tears were healed at 2 years of follow-up, postoperative magnetic resonance imaging showed 69.4% were fully healed and another 25% were partially healed, and 87.5% of patients exceeded patient acceptable symptomatic state (PASS) criteria. Yuen et al.^17^ described the use of a traditional all-inside anchor-based technique for bucket-handle tears and minimized potential complications compared with inside-out techniques.

Here, we describe a technique for repair of large peripheral bucket-handle meniscus tears using an all-inside self-retrieving suture-based device. Early studies have demonstrated successful repair of other tear patterns using the NOVOSTITCH PRO device, including meniscal root tears, horizontal cleavage tears, and radial tears.^13^^,^^18^, ^19^, ^20^, ^21^ All-inside techniques have also been shown to reduce operative time and lower the risk of nerve injury complications compared with inside-out repair.^22^ Furthermore, the NOVOSTITCH PRO device can be used for circumferential compression sutures, which biomechanical studies show reduce gap formation at high cyclic loading and provide the highest load to failure of all repair techniques.^12^^,^^23^^,^^24^

This technique is particularly useful for lateral meniscus posterior horn tears, where the popliteal artery is at significant risk of injury during the procedure (Table 1).^7^^,^^8^ Using an anterolateral portal for traditional all-inside anchor-based repair of lateral meniscus posterior horn tears within 5-10 mm of the root can place the popliteal artery within the direct path of instruments.^5^^,^^6^ Our technique does not violate the capsule, which reduces the risk of injury to the popliteal artery. Use of compression sutures also allows for repair of lateral meniscus tears without risk of capturing the popliteus tendon.^2^ As with other all-inside repairs, this technique risks failure with improperly tensioned suture knots, and as the knots are tied on the mensicus, there is risk of chondral abrasion injury (Table 2).

**Table 1:** Advantages and Disadvantages of the “Under, Over” Technique for Repair of Peripheral Bucket-Handle Tears

Advantages	Disadvantages
Decreased risk of neurovascular injury. Decreased risk of soft-tissue capture (especially popliteus tendon) in repair. Accurate placement of suture through small peripheral tears for repair. Anatomic reduction of bucket-handle meniscus tears. Avoids additional incision for inside-out repair.	Risk of repair failure with poorly tensioned suture knots. Risk of chondral abrasion from suture knots. Increased cost compared with inside-out repair.

**Table 2:** Technique Pearls and Pitfalls of the “Under, Over” Technique for Repair of Peripheral Bucket-Handle Tears

Pearls	Pitfalls
Advance the device into the joint in a low-profile configuration with the lower jaw retracted to minimize risk of traumatic insertion. Retract the lower jaw of device after the peripheral meniscus suture is passed for better maneuverability. Carefully place the second suture to avoid piercing the first suture limb that is now below the meniscus. Use the knot pusher to guide placement of the knot over the peripheral aspect of the tear. An accessory portal can be used to house the suture limbs before tying. Tie sutures after all limbs are passed. Use a 2.0 Kirschner wire to make 3-6 holes on the lateral femoral notch for biological augmentation.	Traumatic insertion of device can damage cartilage. Cartilage damage can occur when maneuvering the device inside the knee. Piecing the initial limb with the second pass will require removal of the suture. Central placement of knots increases the risk of cartilage damage. Suture management is critical. Premature tying of knots can close off access to the peripheral tear. Take care not to damage the ACL fibers.

ACL, anterior cruciate ligament.

The technique presented here to successfully repair large peripheral vertical tears using circumferential compression sutures allows for the peripheral limb of the compression suture to be passed accurately through the peripheral side of the tear with minimal risk to surrounding structures. As compression sutures are increasingly used for various tear patterns, this technique provides a solution that can be applied to all tear patterns in which it is difficult to reach the peripheral meniscus.

## Supplementary Data

ICMJE author disclosure forms

Video 1 This video shows the “under, over” technique for repair of peripheral vertical meniscal tears using the NOVOSTITCH PRO device with circumferential compression stitches. After evaluation of the meniscus with a probe, the NOVOSTITCH PRO device is advanced “under” the central component of the meniscus to pass the first limb of suture through the peripheral side of the tear. The next step is to move the device “over” the central side of the tear and pass the second limb of suture. An arthroscopic crab claw is used to retrieve the peripheral suture limb superior to the meniscus. An arthroscopic knot is tied and cut using a suture cutter. The device can be reloaded with new suture cartridges to place additional circumferential sutures for meniscus reduction.
